# Personalised organs-on-chips: functional testing for precision medicine

**DOI:** 10.1039/c8lc00827b

**Published:** 2018-12-03

**Authors:** Albert van den Berg, Christine L. Mummery, Robert Passier, Andries D. van der Meer

**Affiliations:** a Applied Stem Cell Technologies , University of Twente , Zuidhorst ZH127 , PO Box 217 , 7500 AE Enschede , The Netherlands . Email: andries.vandermeer@utwente.nl ; Tel: +31 53 489 8064; b Anatomy and Embryology , Leiden University Medical Center , The Netherlands; c BIOS/Lab on a Chip , University of Twente , The Netherlands; d Max Planck - University of Twente Center for Complex Fluids , The Netherlands

## Abstract

Organs-on-chips are microfluidic systems with controlled, dynamic microenvironments in which cultured cells exhibit functions that emulate organ-level physiology. They can in principle be ‘personalised’ to reflect individual physiology, for example by including blood samples, primary human tissue, and cells derived from induced pluripotent stem cell-derived cells, as well as by tuning key physico-chemical parameters of the cell culture microenvironment based on personal health data. The personalised nature of such systems, combined with physiologically relevant read-outs, provides new opportunities for person-specific assessment of drug efficacy and safety, as well as personalised strategies for disease prevention and treatment; together, this is known as ‘precision medicine’. There are multiple reports of how to personalise organs-on-chips, with examples including airway-on-a-chip systems containing primary patient alveolar epithelial cells, vessels-on-chips with shapes based on personal biomedical imaging data and lung-on-a-chip systems that can be exposed to various regimes of cigarette smoking. In addition, multi-organ chip systems even allow the systematic and dynamic integration of more complex combinations of personalised cell culture parameters. Current personalised organs-on-chips have not yet been used for precision medicine as such. The major challenges that affect the implementation of personalised organs-on-chips in precision medicine are related to obtaining access to personal samples and corresponding health data, as well as to obtaining data on patient outcomes that can confirm the predictive value of personalised organs-on-chips. We argue here that involving all biomedical stakeholders from clinicians and patients to pharmaceutical companies will be integral to transition personalised organs-on-chips to precision medicine.

## Abstract 2

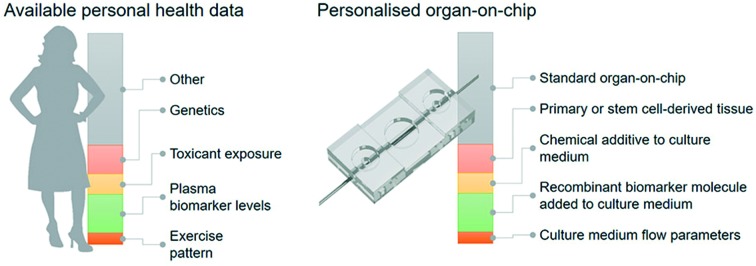
Organs-on-chips can be ‘personalised’ so they can be used as functional tests to inform clinical decision-making for specific patients.

## Introduction

Organs-on-chips are microfluidic cell culture systems with controlled, dynamic conditions that directly emulate the physico-chemical microenvironment of tissues in the human body. As a result, the chips exhibit tissue- and organ-level functions that are not found in other, more simple, *in vitro* cell models. Due to their physiologically relevant read-outs, organs-on-chips are increasingly used for pre-clinical drug testing and in broader areas of biomedical science.^1^ In addition to these applications in pharmaceutical and biomedical research, there is a growing awareness that organs-on-chips can also be regarded as controlled, physical representations of specific patients and therefore be applied directly in the clinic to inform strategies for treatment or prevention of disease. In this review, we will highlight how the controlled integration of person-specific cells, tissue samples and culture parameters based on biometric data enables the development of organs-on-chips that are ‘personalised’, in that they reflect the genetics, physiology and biometric parameters of specific individuals. We will give examples of such personalised organs-on-chips and will highlight how they can contribute to the development and evaluation of treatment strategies for specific groups of patients or individuals.

### Precision medicine uses health data to improve treatment accuracy

The concept of precision medicine, in which each individual would receive tailored treatment for the promotion, maintenance and restoration of their health, is becoming increasingly important in medicine, toxicology, pharmacology and biomedical science due to the increasing recognition of groups of non-responders. This current lack of ‘precision’ in medicine contributes to inefficient healthcare in which many patients receive treatments that are not beneficial for them.^2^ For example, the total number of people that need to take a drug in order for only one of them to benefit from its effects ranges from 5–50 for some of the highest-grossing drugs in the world.^2^ Even more alarmingly, many patients receive treatments that have a negative impact on their health. Millions of people are hospitalised annually due to adverse reactions to their medication, resulting in hundreds of thousands of deaths per year.^3^ By developing methods that can make medicine more precise, ineffective and harmful treatments can be avoided, thus improving quality of life for patients and potentially reducing the cost of healthcare.

The key challenge in precision medicine is to link health-related data of an individual to functional outcomes in their response to specific treatments. The standard approach is to use person-specific data derived from genomics, transcriptomics, imaging, biomarkers and biometrics, followed by longitudinal studies with statistical and computational analysis to identify which patients respond to which treatments. This approach is fruitful and has yielded prime examples of how precision medicine can be applied in practice, for example for selecting person-specific treatments in lung disease and cancer.^4^,^5^

**Fig. 1:**
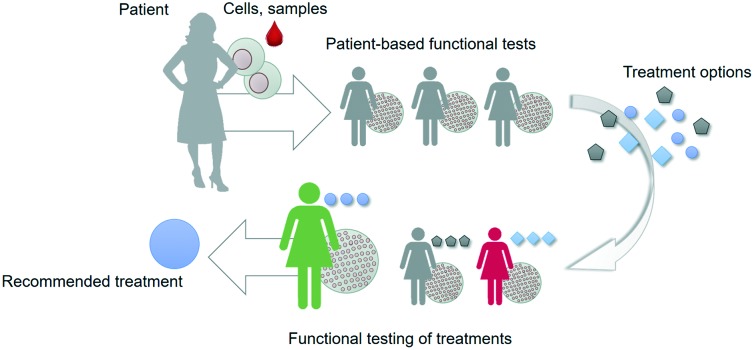
Functional tests can be used to select, optimize or develop treatment options for patients. The tests are based on patient material and offer a functional read-out that is related to an aspect of patient outcome.

However, the current approach typically relies on particular molecular and cellular data points that only capture a very limited subset of the complex web of cellular and tissue-level events that determine outcome in response to treatments. Especially when it comes to developing novel drugs and treatment strategies, data is needed that captures more of the complexity of the disease phenotype of a particular individual. Such an integral approach relies on recapitulating key aspects of the structure and function of patient tissues *in vitro* in a controlled manner, capturing the dialogue between heterotypic cells, followed by assessing the actual functional response of these *in vitro* models to novel treatment strategies; this is referred to as ‘functional testing’ (Fig. 1).^6^,^7^

### Functional testing relies on recapitulating patient complexity in a controlled system

There are already multiple indications of how *in vitro* functional testing can contribute to precision medicine, with notable examples being cultured tumor biopsies for selecting chemotherapy, patient-derived bacterial cultures for selection of antibiotics, and patient-derived organoids for identifying drug efficacy.^6^,^8^–^10^ For example, samples can be isolated from patients who suffer from a bacterial infection. The isolates are then inoculated in various wells of a 96-wells plate that contain a liquid broth as well as a specific antibiotic agent, after which bacterial growth is followed automatically by imaging. By systematically diluting the antibiotic agent in the wells, their mean inhibitory concentrations (MICs) for a specific bacterial isolate can be determined. These MICs are then used to inform the decision of which antibiotic will be effective in treating the patient. Such functional testing is used routinely in the clinic, because genotypic characterisation of bacteria will not always reliably predict their susceptibility and potential resistance to specific antibiotics. In an analogous fashion, primary adult stem cells can be isolated from the rectum of patients with cystic fibrosis and then be grown in a 96-wells plate to form organoids. The function of CFTR, the anion channel affected in cystic fibrosis, can be assessed by tracking the swelling of the organoids in response to the biomolecule forskolin. Organoid swelling correlates with CFTR function, and can therefore be used to evaluate the efficacy of various drugs. Drugs that restore swelling of patient-derived organoids are deemed effective and can then be used to treat the corresponding patient. Such examples demonstrate that the key aspects of functional tests are (1) they capture aspects of the structure and function of tissues in a patient-specific manner; (2) they provide read-outs that are directly related to functional patient outcome; and (3) they are robust, reproducible and compatible with integration in clinical or biomedical research.

The most advanced current functional tests rely on patient stem cell-derived three-dimensional spheroids or organoids, which capture important aspects of the structural complexity of patient tissues. However, these models mostly rely on self-organization of cells with limited control over the tissue culture microenvironment, which means that many aspects of tissue function like transport, metabolism, inflammation, vascularization, contraction, sensing and signalling, are challenging to recreate in a controlled and patient-specific manner. Moreover, their self-organized structure makes it more difficult to design tests that are reproducible, robust and amenable for automation.

### Organs-on-chips capture physiological complexity in controlled, microengineered systems

Organs-on-chips are controlled microfluidic systems in which (human) cells are cultured in engineered microenvironments that recapitulate the essential aspects of tissue geometry, actuation, dynamics, flow and gradients as found in the human body.^11^–^13^ Examples include a “breathing” lung-on-a-chip, circulating (metastatic) tumor cells in networks of perfused blood vessels-on-chips, a gut-on-a-chip with peristaltic actuation and flowing microbes, a multi-organ chip with dynamically cultured liver and pancreas spheroids that maintain glucose homeostasis, and engineered neuromuscular junctions with contracting muscle tissues.^14^–^18^

Organ-on-chip technology offers unique benefits over many current technologies for functional testing, in that it allows a high level of control over biological, physical and chemical cell culture parameters in a single microsystem. This controlled microenvironment promotes realistic tissue physiology and allows integration of relevant functional read-outs. In addition, the small size of organs-on-chips means that they require only small amounts of primary tissue and fluid samples, cells and growth medium, which makes them particularly suitable for standardized, cost-effective manufacturing and automated testing. That aside, however, the key challenge in applying organs-on-chips as functional tests in precision medicine is to ‘personalise’ them so that they are direct reflections of a specific individual.

## Personalisation of organs-on-chips

**Fig. 2:**
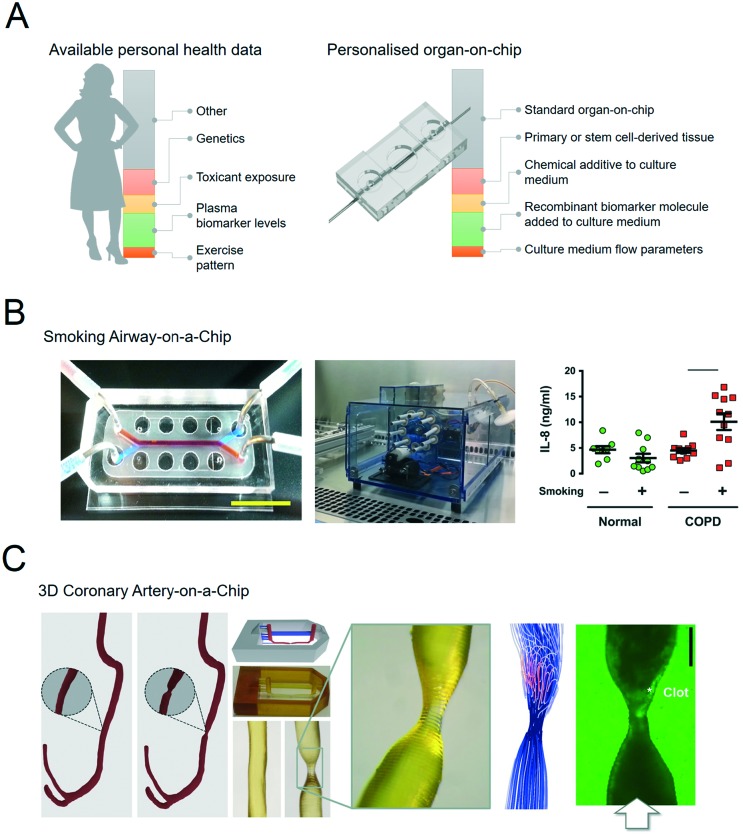
Personalised organs-on-chips can emulate key aspects of a specific person. A, The bar on the left depicts how every individual has a profile of various aspects of their lifestyle, diet, physiology, genetics and environment that determines their predisposition for specific diseases and response to therapeutic interventions. Some of these aspects are unknown (grey), while some have been measured, and represented as ‘personal health data’. Examples are given in red, yellow, green, orange. On the right side, an similar bar depicts the aspects of a person's health data that can be controllably represented in an organ-on-chip system, thus making it ‘personalised’. Such personalised organs-on-chips can potentially be used to make person-specific predictions about disease prevention and treatment. B, Small airway-on-a-chip (left) can be subjected to defined ‘puff’ patterns of cigarette smoking *via* an automated system (middle), to which airway epithelium from COPD patients and healthy controls respond differently in terms of interleukin-8 (IL-8) release (right). Reproduced from Benam, *et al.*^22^ C, Computed tomography data of a coronary artery with and without stenosis (left) can be used to make personalised chips (middle) with unique flow profiles that determine dynamics of thrombosis when the chips are perfused with blood (right). Adapted from Costa, *et al.*^43^

Organs-on-chips can be personalised by using patient samples and health data to engineer aspects of the physical, chemical and biological microenvironment (Fig. 2A).^19^–^21^ An overview of these features with typical examples is given in the following sections.

### Primary tissue, blood and stool samples

The most direct method to develop a personalised organ-on-chip is by including primary samples that have been acquired from patients as biopsies, surplus or waste tissue from surgery, and blood, stool or urine samples.

For example, airway epithelium obtained from lung tissue of patients with chronic obstructive pulmonary disease (COPD) has been used to set up personalised small airway-on-a-chip models.^22^ Using these airway-on-chip models, it could be shown that COPD patients have a different cytokine release profile in response to inflammatory stimuli and cigarette smoke compared to healthy controls (Fig. 2B).^23^

It has also been demonstrated that human blood samples from specific individuals can be perfused through blood vessels-on-chips with ‘generic’ vascular tissue while monitoring platelet aggregation and thrombosis in response to endothelial activation.^24^ Interestingly, blood samples from individuals who were taking anti-platelet medication like aspirin and clopidogrel showed significantly reduced patterns of thrombosis in activated blood vessels-on-chips compared to samples from control subjects, thereby demonstrating the potential to assess drug response of individual patients.^25^ Similarly, when blood vessels-on-chips were perfused with blood samples from patients with sickle cell disease, they exhibited fluctuating blood flow velocities, which is a hallmark of sickle cell disease pathophysiology. Moreover, the fluctuating flow patterns became more stable when the chips were perfused with blood samples from sickle cell patients who were receiving hydroxyurea to treat their symptoms.^26^

Multiple proof-of-concept studies have demonstrated that intestinal bacteria like *E. coli* and *L. rhamnosus* can be co-cultured in dynamic equilibrium with intestinal epithelium in organs-on-chips.^16^,^27^ No studies have as yet reported co-cultured patient-derived microbiota with intestinal tissues in a gut-on-chip but this possibility is widely acknowledged in the field.^28^–^30^

Another potential source of patient-derived primary material is decellularized extracellular matrix. It has been recognized that the extracellular matrix of specific organs and tissues have a strong effect on cells and tissues grown on them. For example, tumor-derived extracellular matrix affects key processes like cell proliferation and migration. There are currently no examples of person-specific or patient-specific extracellular matrices incorporated in organs-on-chips, but multiple examples have demonstrated how to controllably integrate extracellular matrices in organs-on-chips, *e.g.* by local hydrogel patterning or 3D-bioprinting.^31^

### Tissues derived from person-specific stem cells

Because primary patient tissue samples come from an inherently limited source, patient stem cell-based models are a powerful alternative.^32^ Somatic cells of a patient (*e.g.* skin fibroblasts, blood-derived mononuclear cells, or urine-derived renal epithelial cells) can be re-programmed to a pluripotent state by transient expression of four transcription factors. The resulting human induced pluripotent stem cells (hiPSCs) can in principle differentiate to any tissue of the human body while remaining genetically identical to the original donor. Similarly, adult human stem cells can be isolated from donor tissue and be differentiated in the lab into the specific tissues from which they originate, with skin, intestinal and gastric adult stem cells being notable examples. Due to their inherent proliferative potential, both hiPSCs and adult stem cells offer researchers an unlimited source of patient-derived material. Moreover, stem cell technology offers unique advantages, such as the reproducible production of stable batches of cells by implementing robust differentiation protocols, and the genetic modification of stem cells to generate specific genetic reporter lines, genetic knock-outs and isogenic control lines. Many examples exist of *in vitro* disease models based on complex hiPSC-derived tissues, and the field is growing rapidly.^33^ The differentiation of hiPSC into specific cells or tissues is a major technological challenge, and protocols to generate stable sources of cells are constantly being developed and updated. Still, an inherent challenge of cells differentiated *via* these protocols is that they are usually immature or ‘fetal’ in their gene expression patterns and functional phenotype, which limits their relevance as a direct representation of the person from which they were derived.^32^ Moreover, the reprogramming of patient cells into a pluripotent state means that they will no longer capture cumulative environmental and age-related aspects of a disease.

Despite these limitations there are already multiple examples of organ-on-chip systems that incorporate hiPSC-derived or adult stem cell-derived cells and tissues, such as blood vessels-on-chips, a blood–brain barrier-on-chip, heart-on-chip and gut-on-chip systems.^34^–^38^ The first examples of patient-specific organs-on-chips based on patient-derived hiPSC-derived cells have also been described. For example, microengineered blood vessels based on cells derived from hiPSC of Hutchinson–Gilford progeria syndrome patients exhibited exacerbated inflammation as well as reduced vasoactive function.^39^,^40^

### Biomarker levels

Biomarkers are biological molecules or cells that can be isolated from a patient and then quantified in the laboratory to diagnose or track diseases or disease-related processes. Ideally, biomarkers have a high sensitivity and specificity, which means that a biomarker – or a particular level of a biomarker – is found in all or most patients, but not or hardly ever in non-affected individuals. Most biomarkers, such as troponin in cardiac injury, are simply downstream effects of a disease process. However, some biomarkers are more directly and causally involved in disease pathophysiology. Integrating such pivotal biomarkers in organ-on-chips systems at the same levels as measured in a specific patient allows the controlled engineering of patient-specific organs-on-chips, without having to directly include patient-derived samples. Molecular biomarkers are typically small biomolecules such as RNAs, peptides and proteins, that can also be produced in the laboratory. As such, the recombinant or engineered versions of biomarkers can be added to cell culture media for organ-on-chip systems to mimic specific integral aspects of a disease.

There are a number of examples of how the controlled integration of biomarkers in organs-on-chips can be used to mimic patient phenotypes. First of all, in a small airway-on-a-chip model, many aspects normally associated with asthma, such as increased excretion of inflammatory cytokines and reduced pulmonary epithelial ciliary beating frequency, could be induced by controlled treatment of ‘healthy’ chips with the cytokine interleukin-13 (IL-13).^22^ IL-13 is accepted as a central player in asthma: its expression can be induced directly in patients after low-dose allergen provocations, and it is used as a clinical biomarker to evaluate treatment efficacy.^41^–^43^ A second example is provided in a study that revolved around the use of a blood vessel-on-chip system to detect thromboembolic side-effects of a (now obsolete) therapeutic monoclonal antibody against CD40 ligand (CD40L/CD154).^44^ The pro-thrombotic effect could only be detected when normal blood samples were spiked with soluble CD40-ligand (sCD40L) at levels that are typically only found in patients with auto-immune disease or viral infections. sCD40L is considered to be a pro-inflammatory biomarker in those patients.

### Biomedical imaging data

Biomedical imaging is a routine part of diagnosing pathologies, informing treatment strategies and evaluating treatment response. Biomedical imaging is becoming increasingly more sophisticated, with a strong increase in modalities that not only visualise the structure of a tissue or organ, but also its function in terms of blood flow, oxygenation, transporter properties, *etc.* This rich source of clinical data can in principle be used to design personalised organs-on-chips in which geometries, flow parameters, oxygen levels and other culture parameters are based on patient imaging data. We have recently published an example of this approach in which computed tomography angiography data of a coronary artery was used to engineer a 3D-printed microfluidic chip that was subsequently lined by human endothelium and perfused with human blood (Fig. 2C).^45^

### Biometric data

Apart from the specific examples mentioned in the previous sections, there is considerable person-specific data related to bodily functions, environment and lifestyle that can in principle be used to design personalised organs-on-chips. Some examples of this ‘biometric’ data are dynamic heart rate patterns, patterns of exercise, sunlight exposure, air quality, patterns of smoking, substance abuse, diet and blood glucose levels. An increasing amount of this type of person-specific, health-related data is being collected; not just in a strictly clinical context, but also by wearables and mobile devices, which are collectively known as mobile health, or ‘mHealth’, products.

This source of biometric data has currently not been used to systematically engineer personalised organs-on-chips, but it is clear that many of the aforementioned biometric parameters can in principle be incorporated in organ-on-chip systems. For example, organ-on-chip systems have been exposed to various patterns of cigarette smoking to examine small airway inflammation patterns (Fig. 2B); microengineered heart tissues have been subjected to different ‘heart rates’ by pacing them with external electrodes; microfluidically connected cultures of hepatic and pancreatic microtissues have been exposed to various external glucose levels to evaluate the regulatory, homeostatic response.^17^,^23^

## Implementing personalised organs-on-chips in precision medicine

The potential for personalising organs-on-chips is becoming evident and from the examples above it is clear that features such as soluble markers, genetics, geometries and flow patterns can all be tailored based on person-specific data. However, personalised organ-on-chip systems have not yet been applied in driving personalised treatment decisions in the context of precision medicine. Key challenges for successful implementation are related to (1) obtaining personalised health data, that includes addressing any ethical issues, (2) confirming results from personalised organs-on-chips by comparing them with symptoms, drug responses and outcome in patients, (3) mimicking aspects of multifactorial and chronic diseases in organs-on-chips, and (4) implementing the technology in a cost-effective manner.

### Involving biomedical stakeholders will be essential for application of personalised organs-on-chips in precision medicine

Organ-on-chip development is interdisciplinary, with engineers and biologists collaborating to design and build systems that recapitulate organ-level physiology. With the move towards personalised organs-on-chips for precision medicine, new groups of stakeholders are needed in the development process, particularly biomedical scientists, clinicians and patients. Patients and clinicians will need to provide the tissue samples as well as relevant personalised health data, taking into account ethical aspects like proper consent, data protection and ownership. Moreover, biomedical stakeholders are required to translate the promising findings (*e.g.* candidate gene variants, biomarkers, metabolites, dietary supplements) from their clinical studies into specific aspects of an organ-on-chip that should be personalised. This input will be essential in the technical development of organ-on-chip systems, as it determines what level of complexity in terms of cell types, microenvironment parameters, tissue interactions, is required and which read-outs, assays or sensors need to be included to evaluate relevant physiological effects in the system.^46^

Biomedical stakeholders will also play an essential role in providing longitudinal data to confirm or refute the health-related predictions from personalised organs-on-chips. This will be the main method to improve personalised organ-on-chip technology and to identify specific domains of precision medicine in which the technology will have added value. Confirmation of functional results from organs-on-chips can be facilitated by implementing read-outs that are analogous to those used in clinical settings.^46^ For example, measuring clinical biomarker levels or applying imaging methods like optical coherence tomography, ultrasound and micro-computed tomography to analyse a functional response in an organ-on-chip system could enable virtually direct comparison to patient health data that has been obtained with similar techniques.

### Further biotechnical development and upscaling of personalised organs-on-chips is necessary

Current organs-on-chips are mostly applied in drug development and biomedical research, to evaluate toxicity and efficacy of drug candidates and to understand mechanisms of disease.^47^ These application domains are also the main markets targeted by companies in the field. However, the implementation of organs-on-chips in precision medicine will add new requirements for organ-on-chip technology. First of all, large numbers of personalised organs-on-chips will have to be produced to confirm that their data indeed has predictive value for the persons that they are supposed to emulate. In addition, it should be practically feasible and cost-effective to make personalised organs-on-chips in a short time-period so they can be used to inform clinical decisions regarding treatment or prevention strategies. Depending on the level of personalisation, this means that major progress will have to be made in optimising stem cell-differentiation protocols, primary sample processing, customised device production, and automated software to adjust culture parameters per individual chip. The economic challenges associated with cost-effective engineering of personalised organs-on-chips will be formidable, because of their inherent complexity and the integration of patient-derived samples. Multiple cell types from various tissues or organs, as well as tissue-specific dynamic culture parameters will have to be included and customised based on personal health data to capture the essential aspects of a complex disease phenotype. Because the development of organ-on-chip technology is so recent, no systematic academic literature exists yet on the economic impact of applying organs-on-chips in pharmaceutical settings, let alone in clinical functional testing. Early, non-peer reviewed literature on the economic impact of implementing organs-on-chips in drug development suggest that doing so would lead to cost savings.^48^,^49^ However, this statement is only based on a small sample of expert interviews, expert review articles, or on hypothetical scenarios of optimizing drug development. For a more comprehensive, quantitative assessment of the economic impact of implementing organs-on-chips as functional tests in the clinic, it will be essential to get an estimate of the savings associated with avoiding unnecessary patient treatment, as well as a realistic estimate of costs (*e.g.* based on the pricing of commercial organ-on-chip systems, combined with typical costs per case for biobanking of patient samples).^50^ Future cost savings can potentially be achieved by collaboration with established biobanking initiatives to obtain relevant tissue samples. Moreover, it may be feasible to use a single personalised organ-on-chip as a ‘representative’ for multiple individuals within a specific patient subgroup instead of developing personalised organs-on-chips for each individual. Finally, cost reduction may come from further standardisation, miniaturisation and parallelisation of organ-on-chip culture systems. There are already multiple examples of how to parallelise organ-on-chip systems. Some of such solutions are monolithic platforms with a small footprint that build on existing cell culture standards, *e.g.* by engineering many microfluidic chips in a plate with the footprint of a single conventional wells-plate, or of a single microscope slide, thereby taking full advantage from the compatibility with existing laboratory equipment like pipetting robots and microscopes.^51^,^52^ In contrast, other efforts towards parallelisation are based not on single devices with many side-by-side chips, but instead rely on a modular platform with individual organ-on-chip building blocks and standardised connections to a holder or pump system. Such platforms allow flexible integration of multiple chip modules, but are often quite large due to the interfaces of many individual chips. The main advantage of a modular platform is that it would in principle allow parallelised development and exchange of individual modules, including ones that add system functionality like pumping, sensing and analysis. However, for the free exchange of modules to become a reality, it will be important for labs and companies to not rely solely on in-house standards, but instead to develop common standards for microfluidic chips to interface with each other. Stakeholders from the field are attempting to define such standards *via* International Organization for Standardization (ISO) documentation.^53^

## Outlook

In the coming years, the concept of personalised organs-on-chips will likely progress from its current position in academic proof-of-concept studies to confirmation in the context of precision medicine. If it can indeed be demonstrated that personalised organs-on-chips have added value in directing personalised treatment and prevention strategies, they are likely to become an integral part of broader advances in medicine towards a discipline that is increasingly predictive, preventive, personalised and participatory (‘P4 medicine’).^54^ With the recent advent of methods for collecting health-related data (-omics, mHealth), strategies for treatment and prevention will become increasingly person-specific and will not just be based on broad demographics or symptoms. This transition should lead to more effective healthcare, reduce time and cost of drug development, and avoid casualties related to side-effects. Personalised organs-on-chips can play unique roles in effecting this transition because they provide a physical system to experimentally optimise new strategies for treatment and prevention, thereby enriching all the health data that is currently considered to be the primary source of person-specific treatments.

**Fig. 3:**
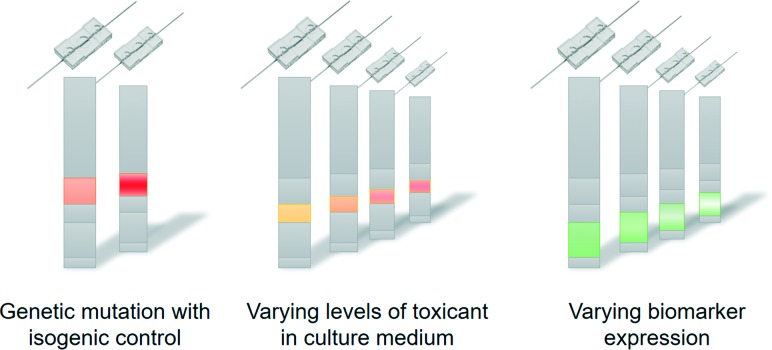
Personalised organs-on-chips can be used to understand how particular health-related parameters affect functional outcome of an individual. Specific aspects like genetic variation (left), exposure to toxicants (middle) or grades of particular disease processes (right) can be systematically varied in different organs-on-chips while all other relevant and personalised aspects are held constant. This approach allows the ‘reverse engineering’ of the interactions that generate the functional outcome for an individual.

Technically, the personalisation of organs-on-chips is currently limited by the availability of health data and person-derived tissue samples, as well as by a relatively low-throughput and sub-optimal automation of organ-on-chip fabrication, tissue culture and system operation. As the technology matures, it is likely that many aspects of single organ-on-a-chip systems will become personalised. In that case, the controlled formats and biology of organs-on-chips will make it possible to ‘reverse engineer’ how health-related parameters (exercise patterns, diet, genetics) interact to affect personal health (Fig. 3).^55^ For example, if a large-scale genomic screening has led to the identification of genetic variants that are associated with disease, then such variants can be repaired or introduced by gene editing through CRISPR/Cas9 in personalised organs-on-chips of specific patients or non-patients. This allows the systematic evaluation of the role of a genetic variant in the disease mechanisms by comparing ‘mutated’ organs-on-chips directly with their isogenic control counterparts (Fig. 3, left). Similarly, population studies may point to significant contributions of diet (or other factors of lifestyle or environment) in increasing the risk of a disease. Personalised organs-on-chips can be used as experimental systems to systematically study the functional effects of varying levels of specific dietary factors, while keeping all other relevant aspects (like genetics, systemic inflammation) constant between conditions. This approach of first setting up a personalised organ-on-chip that includes a disease-related parameter as identified by observational studies, followed by an experimental functional comparison with matched, person-specific control organs-on-chips will lead to greater understanding of disease mechanisms, which in turn may lead to identification of new drug targets and biomarkers.

Finally, the dynamic nature of organ-on-chip systems may in the future make it possible develop organs-on-chips in which cell culture parameters are controllably altered as new or updated health data becomes available over time. This may be important when analysing the effectiveness of lifestyle changes for prevention. Such dynamic organ-on-chip systems may even lead to personalised organs-on-chips and body-on-chip systems that are always ‘online’, meaning that their culture conditions are continuously updated based on newly available health data. Such ‘avatars’ could either exist inside a ‘pod’ in a laboratory, or even be connected to a person's body as an extracorporeal device like the Badimon chamber or an insulin pump. With proper online read-outs and sensors, the systems could then serve as ‘sentinels’ that make predictions about health-related events as they are being exposed to similar conditions as the person wearing them, reminiscent of how canaries were used in the 20th century to signal dangerous levels of carbon monoxide in coal mines.

## Conclusions

Organs-on-chips are well-suited to capture personal health-related parameters in a controlled system, while providing functional read-outs that are directly linked to tissue-level and organ-level physiology. The first examples of personalised organs-on-chips have appeared in literature, with a current focus on integration of person-derived blood samples, primary tissue cells and stem cells. However, other examples of how to integrate personal health-related parameters in organs-on-chips are also appearing, with examples in geometry, flow and dynamic exposure to toxicants. By engaging biomedical stakeholders in further development of personalised organs-on-chips, the implementation of this technology in precision medicine will likely be a matter of time. Finally, because organs-on-chips are compact and have a controlled fluidic circuitry, they might in the future even be kept ‘online’ permanently, either in the lab or by connecting them to the body of their personal hosts.

## Conflicts of interest

The authors declare no conflicts of interest.
